# Orthopedic Surgeons’ Perspectives on the Decision-Making Process for the Use of Bioprinter Cartilage Grafts: Web-Based Survey

**DOI:** 10.2196/14028

**Published:** 2019-05-15

**Authors:** Àngels Salvador Verges, Luis Fernández-Luque, Francesc López Seguí, Meltem Yildirim, Bertran Salvador-Mata, Francesc García Cuyàs

**Affiliations:** 1 Digital Care Research Group Universitat de Vic - Universitat Central de Catalunya Barcelona Spain; 2 Qatar Computing Research Institute Hamad bin Khalifa University Qatar Qatar; 3 TIC Salut Social Generalitat de Catalunya Mataro, Barcelona Spain; 4 Department of Experimental and Health Sciences Centre for Research in Health and Economics Universitat Pompeu Fabra Barcelona Spain; 5 Centre for Health and Social Care Research Faculty of Health Science and Welfare Universitat de Vic - Universitat Central de Catalunya Vic Spain; 6 Research group on Methodology, Methods, Models and Outcomes of Health and Social Sciences Vic Spain; 7 University Pompeu Fabra Barcelona Spain; 8 Hospital Sant Joan de Déu Digital Care Research Group Universitat de Vic - Universitat Central de Catalunya Barcelona Spain

**Keywords:** orthopedic surgeons, online survey, 3D bioprinting, cartilage, graft

## Abstract

**Background:**

Traumatic and degenerative lesions in the cartilage are one of the most difficult and frustrating types of injuries for orthopedic surgeons and patients. Future developments in medical science, regenerative medicine, and materials science may allow the repair of human body parts using 3D bioprinting techniques and serve as a basis for new therapies for tissue and organ regeneration. One future possibility is the treatment of joint cartilage defects with in vivo 3D printing from biological/biocompatible materials to produce a suitable cell attachment and proliferation environment in the damaged site and employ the natural recovery potential of the body. This study focuses on the perspectives of orthopedic surgeons regarding the key factors/determinants and perceived clinical value of a new therapeutic option.

**Objective:**

This study aimed to determine the knowledge and expectations of orthopedic surgeons regarding the clinical use of bioprinted cartilage.

**Methods:**

The survey, conducted anonymously and self-managed, was sent to orthopedic surgeons from the Catalan Society of Orthopedic and Traumatology Surgery. In accordance with the method devised by Eysenbach, the Checklist for Reporting Results of Internet E-Surveys was used to analyze the results. The following factors were taken into consideration: the type and origin of the information received; its relevance; the level of acceptance of new technologies; and how the technology is related to age, years, and place of experience in the field.

**Results:**

Of the 86 orthopedic surgeons included, 36 believed the age of the patient was a restriction, 53 believed the size of the lesion should be between 1 and 2 cm to be considered for this type of technology, and 51 believed that the graft should last more than 5 years. Surgeons over 50 years of age (38/86, 44%) gave more importance to clinical evidence as compared to surgeons from the other age groups.

**Conclusions:**

The perspective of orthopedic surgeons depends highly on the information they receive and whether it is specialized and consistent, as this will condition their acceptance and implementation of the bioprinted cartilage.

## Introduction

### Background

The cartilage is a specialized connective tissue that does not contain nerves, blood, or lymphatic vessels and is formed by the differentiation of mesenchymal cells. It is flexible and composed mainly of extracellular matrix that contains chondrocytes. Defects on the articular cartilage do not heal spontaneously and tend to develop into osteoarthritis, which in turn alters the articular function and can cause disability and progressive loss of quality of life [1]. The exact incidence of symptomatic lesions of cartilage in the general population is unknown. In some large epidemiological studies, cartilage lesions have been observed in 5%-11% of diagnostic arthroscopies in predominantly young adults with joint pain [2]. However, injuries are often focal chondropathies, resulting in matching injuries on the opposing surface such as in meniscus or ligament injuries to the knee. They are also related to misalignments of the axis articulating load. Two types of techniques are used for the treatment of symptomatic lesions of cartilage: the reconstructive techniques have been in use since the 1950s and the regenerative techniques are newly introduced in tissue engineering [3]. Current surgical procedures [4] such as microfracture surgery, mosaicplasty, and allografting have limited efficacy [5], and none of them are significantly more successful than the others [6]. More innovative surgical treatments from the past few years, including autologous chondrocyte implantation and matrix-induced autologous chondrocyte implantation [7], which require a previous surgery to obtain the cells, have markedly improved the outcomes of chondral defect treatments [8] but often, the resulting repairing tissue is of low performance, and surgery only delays the onset of degeneration and osteoarthritis [8].

The development of regenerative medicine and tissue engineering–oriented techniques may contribute to the knowledge of the field of joint injuries. This, in turn, could lead to better articular disease treatment techniques and resolve the clinical problem of healing critical size articular osteochondral defects [9].

The key elements of tissue engineering are tissue-forming cells, structural scaffolds, and signaling molecules, the combination and application of which result in a functional tissue construct to promote tissue healing and regeneration [10]. Tissue engineering strategies typically aim to homogenously distribute biological factors such as cells and growth factors throughout a biomaterial matrix [11].

Autologous chondrocytes could be an obvious choice for regeneration of articular cartilage injuries. However, traditional treatments based on chondrocytes have identified several drawbacks of such chondrocytes: (1) they have a low rate of proliferation; (2) although it is easy to isolate them, the number of obtainable cartilage cells is limited; and (3) there are implications for morbidity of the donor site. Consequently, the use of other cell types for different tissue engineering applications such as stem cells [12] (embryonic, mesenchymal, cord blood stem cells, obtained from adult tissue, or induced pluripotent stem cells [13]) are the future for treatment.

Regenerative medicine [14] and tissue engineering [15] are current approaches aimed to solve these problems. These new possibilities could drive the paradigm shift from symptomatic treatment [16] in the 20th century to healing treatment of the 21st century [17,18].

Bioprinting [19], defined as a real-time disposition of structural biomaterials and live cells to create tissues and organs that imitate the characteristics of the injured tissue/organ, is moving forward very quickly, but because the process of obtaining tissues by using this technique depends on many factors, several technological needs must be met beforehand.

In an animal study, Di Bella and collaborators [20] used a 3D printer of a hand *in situ*. 3D printing, an innovative bioprinting technology, allowed the surgeon to use tissue engineering techniques at the time and place of need, using the hand-held printer Biopen. This instrument, with separate cartridges for each biomaterial, allows the surgeon to set the different layers right on the cartilage. Furthermore, it does not require a computed tomography scan, as the surgeon can use the Biopen directly where it is needed [21]. No studies in humans have been published, but according to the authors, it will not take long for the results to be obtained. As researchers develop bioprinted grafts, the knowledge of clinician priorities will facilitate their refinement and implementation.

Implementation research [22] seeks to resolve a wide range of issues found in the process of clinical application. The goal is to understand how and why the researchers’ new suggestions are understood in the clinical sphere and find the best approaches to develop them [23]. In previous research [24] focusing on orthopedic surgeons, the information aspect was highlighted. Therefore, this study will focus on the information received, with the aim of understanding the surgeons’ process of decision-making and to identify their expectations regarding the ideal bioprinted cartilage graft.

### Aim

This study aims to understand how orthopedic surgeons make clinical decisions and to assess their knowledge and opinion on this topic as well as their needs concerning the bioprinted cartilage graft.

Two main goals were set: (1) to obtain a better understanding of the orthopedic surgeons’ decision-making process, and, by using this knowledge, to understand which factors would drive surgeons to use the graft on the treatment of cartilage lesions and (2) to identify surgeons’ expectations regarding the use of grafts and the key factors to be addressed for surgeons to consider implantation of such grafts on a patient. Hence, the overall purpose was to define the ideal scenario and characteristics of the graft for successful implantation.

## Methods

### Contextualization of Research and Ethical Approval

This survey and its questions were defined in the context of both a previous qualitative study [24] about the barriers and facilitators in the clinical use of bioprinted cartilage, which had derived conclusions from semistructured interviews and focus groups with orthopedic surgeons, and a literature review. Both these approaches helped identify the most pressing issues, on which this survey was focused.

Approval was obtained from the Committee of University Research from a University of Vic - University Central of Catalonia (registration number 28/2017).

Participants of the study were informed that the survey was anonymous and notified about the average completion time, and all information mentioned in the survey was credited to its source. The results were stored on a university-owned website, with private access for the authors.

### Survey Design

Before the final version of the survey was sent, a trial version was sent to 17 orthopedic surgeons to ensure that both the subject and the instructions were understood and to measure the average time for completion. After the results were obtained, some changes were introduced in the survey: questions that asked to rate agreement were scored on 10 possible scores instead of 5 possible scores, adding more options; the writing of the questions and answers was edited; and more specific questions were added.

The final questionnaire, with 25 self-managed questions, allowed identification of the decision-making patterns of orthopedic surgeons. The constituent elements of the questionnaire are configured in five domains: (1) demographic questions, (2) information and knowledge of 3D printing, (3) knowledge about the graft’s qualities, (4) knowledge about the expectations for this new technology, and (5) scientific evidence and clinical trials.

### Sample Population

The Col·legi de Metges de Barcelona (the Medical Association of Barcelona) has 1081 currently active orthopedic surgeons registered, including 906 men (83.8%) and 175 women (16.2%). Of the members, a total of 849 also belong to the Catalan Society of Orthopedic and Traumatology Surgery (SCCOT), which is a nonmandatory affiliation. An email was written to all SCCOT surgeons, asking them to participate in the study, with a survey link. Of these, 72 emails were returned to the sender (the email address was wrong), and 777 orthopedic surgeons received the email.

In Spain, orthopedic surgeons can develop their work in the public and private sector at the same time. The specialization in sports orthopedics is not separately regulated. Because the survey was anonymous, we could not determine how many of the respondents were from the academic field.

The survey was voluntary, had no incentives, consisted of only one page, and allowed for review of the answers before sending. Answers could be easily obtained and homogenized, as they were in the same order, level, and presentation within all instances, which reduced the error margin and facilitated data retrieval.

The response rate was 11% (86/777). The average time to completion was 15.43 minutes. The survey was kept active until a sufficient number of answers were collected.

The calculation of the size of the finite sample was obtained using the Murray and Larry [25] formula. Configured with a margin of error of 10%, a confidence level of 95%, and a population of 777, the resulting sample size was 85.57 people.

### Statistical Analysis

IBM SPSS Statistics software for Windows, version 21.0 (IBM Corp, Armonk, New York), was used to analyze the answers of the survey. The Kolmogorov-Smirnov test was used to evaluate the homogeneity of the data. A descriptive analysis with the distribution of frequencies, averages, and SDs was conducted. A comparative analysis was conducted using the Kruskal-Wallis test, Mann-Whitney *U* test, and Chi-squared distribution. The results have been presented following the Checklist for Reporting Results of Internet E-Surveys [26].

## Results

The results have been categorized into two blocks: the information that affects the decision-making process of orthopedic surgeons and the qualities that a graft should ideally have, to be implanted in patients.

### Demographic Data

Participants were first asked about their gender, age, experience, and type of hospital where they practiced surgery (Table 1).

Hospitals in Spain can be classified as low, medium, or high complexity depending on the type of technology they use and the type of medical assistance they offer. Research and teaching are conducted at medium- and high-complexity hospitals.

### Information Linked to the Decision-Making Process of Surgeons

The main aspect of implementation research is evaluating and determining the level of information specialists need to acquire in order to implement the new technology. To obtain a better understanding of the information orthopedic surgeons depend upon to make decisions, answers have been classified by type and origin of the information, relevance, level of acceptance of new technologies, and how the technology is related to age, place, and years of experience.

### Information Received

Participants were asked if they had received any type of information related to new medical applications and 3D printing (Table 2).

Almost 70% of the surveyed participants reported that they have received information related to new medical applications and 3D printing via any medium. They considered themselves updated in the medical applications of new technologies as per their own perception.

**Table 1:** Demographic data of the participants (N=86).

Demographic		Surgeons, n (%)
**Gender**		
	Women	32 (37)
	Men	54 (63)
**Age**		
	<40 years	27 (31)
	40-50 years	14 (16)
	>50 years	45 (52)
**Years practicing medicine**		
	5-15 years	33 (38)
	15-30 years	28 (33)
	>30 years	25 (29)
**Performing surgical activity**		
	Yes	80 (93)
	No	6 (7)
**Work placement**		
	Hospital of low complexity	16 (19)
	Hospital of medium complexity	34 (40)
	Hospital of high complexity	36 (42)

**Table 2:** Information received and new technologies updates.

Source of information		Value
**Have you received any kind of information, through any means, about the latest progress on 3D printing? n (%)**		
	Yes	60 (70)
	No	26 (30)
**Do you consider yourself up to date regarding new 3D printing technologies emerging in the medical field?**		
	Reported scores (range)	2-10
	Mean (SD) score	6.88 (1.66)

### Information Relevance

From the previous question, the relevance of the information was analyzed. Relevance was determined by the effect information had in making surgeons feel more and better informed.

To evaluate the relevance, the source of information was analyzed. We analyzed whether those who had received information (by any medium; n=60) considered themselves better informed than the rest and whether those who had received information from specialized companies (n=20) had more knowledge than those who had not received any such information (Table 3).

Crossing the two variables from the previous Table 3 showed that participants who received information by any means considered themselves more knowledgeable (*P*=.001) than those who had not received any kind of information. Of the former, those who had received information from companies who are developing these technologies perceived their knowledge to be higher than that of the rest (*P*=.006). In addition, participants informed by specialized companies showed higher self-perceived knowledge (mean 6.95, SD 1.76) than those who received information via other sources (mean 6.27, SD 1.99).

Regarding the bioprinted cartilage graft specifically, participants who received information on the medical application of 3D printing (n=60) and considered themselves informed (mean 6.27, SD 1.99) were asked how specific and from which source the information they had received on bioprinted cartilage was (Table 4).

Of the 60 participants who received general information on 3D printing, only 27 (45%) knew about bioprinted cartilage, and the information had been acquired from their colleagues (18%) or the scientific literature (27%).

**Table 3:** Level of self-perceived knowledge on 3D printing in relation to the information received (N=86).

Source of information		Surgeons, n (%)	Mean (SD)	*Z*^a^	*P* value
**Have you received any kind of information, through any means, about the latest progress on 3D printing?**				**–3.225**	**.001^b^**
	Yes	60 (70)	6.27 (1.99)		
	No	26 (30)	4.69 (1.73)		
**Have you been informed by a specialist company about 3D technology?**				**–2.746**	**.006^b^**
	Yes	20 (23)	6.95 (1.76)		
	No	66 (77)	5.44 (2)		

^a^Mann-Whitney *U* Test.

^b^*P*<.01.

**Table 4:** Means of learning about bioprinted cartilage among participants who received information on 3D printing (Question: If you have received any 3D printing information regarding bioprinted cartilage, through which channel was it? N=60).

Means of learning	Surgeons, n (%)
Through other colleagues	11 (18)
I read a lot of new research	16 (27)
I read a little new research	2 (3)
I have no information about it	30 (50)
I’m not interested in it	1 (2)

### Level of Acceptance of New Technologies

To determine if there was a relationship between the relevance of the information received and the acceptance of new technologies, we analyzed the acceptance level of the bioprinted cartilage graft among participants who had higher self-perceived knowledge (20/86) and had received the information from specialized 3D companies (Table 5).

Regarding the perception or ease of acceptance of the bioprinted cartilage graft, there was a significant difference between participants who were informed by specialized companies and those who were not (*P*=.02). The more information the participants had, the higher was the level of acceptance. Of those who had not received any specific information, no significant conclusion could be deducted (*P*=.08).

### Relation to Demographic Data

To define if the process of decision-making by orthopedic surgeons could be linked to their demographic data, three variables were analyzed: age, years of experience, and place of experience (Table 6).

Considering self-perceived knowledge, the only difference identified was in the age of the participants. Participants aged over 50 years (38/86) considered themselves to be significantly more informed on new technologies than those of other age groups (*P*=.05). No differences were observed regarding the place and years of experience.

**Table 5:** Surgeons’ acceptance of the use of bioprinted cartilage grafts for their patients, according to the source of information (Question: If the researchers/biotech industry could give us a cartilage graft made with bioprinting, would you think about the convenience of using it in your patients? N=86).

Source of information		Surgeons, n (%)	Mean (SD)	*Z*^a^	*P* value
**Have you been informed by a specialized company about 3D technology?**				**–2.254**	**.02^b^**
	Yes	20 (23)	8.40 (1.53)		
	No	66 (77)	7.53 (1.69)		
**Have you received information, through any means, about the latest progress on 3D printing?**				**2.736**	**.75^b^**
	Yes	60 (70)	7.65 (2.38)		
	No	26 (30)	7.92 (1.41)		

^*a*^Mann-Whitney *U* test.

^b^*P*<.05.

**Table 6:** Influence of demographic data of orthopedic surgeons on the knowledge of new technologies (Do you consider yourself up to date regarding new technologies emerging in the medical field? N=86).

Demographic		Surgeons, n (%)	Mean (SD)	*X*^2a^	*P* value
**Age**				**3.6**	**.05^b^**
	<40 years	28 (33)	6.5 (1.79)				
	40-50 years	20 (23)	7.2 (1.85)				
	>50 years	38 (44)	7 (1.45)				
**Years practicing medicine**				**3.5**	**.18**
	5-15 years	33 (38)	6.42 (1.88)				
	15-30 years	28 (33)	7.14 (1.55)				
	>30 years	25 (29)	7.2 (1.38)				
**Work placement**				**0.07**	**.96**
	Hospital of low complexity	16 (19)	6.94 (1.48)				
	Hospital of medium complexity	34 (40)	6.91 (1.65)				
	Hospital of high complexity	36 (42)	6.83 (1.79)				

^a^Kruskal Wallis test.

^b^*P*<.05.

### Analysis of the Qualities of the Archetypal Bioprinted Cartilage Graft

The second goal of this study was to identify the qualities of the ideal cartilage graft for application by the orthopedic surgeons in relation to patient characteristics, type of lesion, and perceived difficulties of their use.

### Factors Determining the Ideal Graft

The essential characteristics of the bioprinted graft that were analyzed to identify the suitable age of the patient for the implantation, ideal size of the lesion, and duration of the graft. Participants were also asked to choose the most relevant of five suggested qualities (Table 7).

Regarding the age of the patient, 50% of the participants would not implant the graft on patients aged over 70 years, whereas 42% of them did not consider age to be a delimitating factor. Most of them (62%) considered the ideal size of the injury to be between 1 and 2 cm for implantation of a bioprinted cartilage graft. However, 27 (31%) of participants would consider such grafts for lesions over 3 cm. Almost all participants would reject a graft that lasted less than a year. Moreover, 51 of them (59%) said they would not recommend the graft to the patient unless it lasted more than 5 years.

Of the suggested qualities, the two most often selected (78%) were duration of the graft and patient safety (no side effects to general health). One less-often selected quality was ease of implantation, only considered by 50% of the participants.

### Perceived Difficulties

The link between the perception in relation to the difficulties and the type of hospital was examined to determine if perceived difficulties were related to surgeons’ place of work or whether it was the individual perception of the orthopedic surgeon (Table 8).

Table 8 shows the number of answers depending on the type of hospital and the percentage that each subpopulation represents in relation to the type of hospital.

The main difficulties considered by orthopedics in low-complexity hospitals were outcome uncertainty (ie, lack of clinical trials that prove successful outcomes) and authorization issues by the hospital management. In medium-complexity hospitals, surgeons shared these worries, although to a lesser extent. In high-complexity hospitals, however, the main issue was patient safety, followed by outcome uncertainty.

**Table 7:** Determining factors of the archetype graft (N=86).

Factor		Surgeons, n (%)
**To what extent do you consider the patient's age to be a limitation in the use of bioprinted cartilage?**		
	I do not see any age limitation	36 (42)
	Under 20 years of age	7 (8)
	Over 70 years of age	43 (50)
**To what extent do you consider the size of the cartilage injury to be a limitation?**		
	<1 cm	6 (7)
	1-2 cm	53 (62)
	>3 cm	27 (31)
**What minimum duration would the implant need to have for you to recommend it to your patients?**		
	<1 year	6 (7)
	1-5 years	29 (34)
	>5 years	51 (59)
**What are the most significant variables that you ask for in a bioprinted cartilage, before deciding to use it on your patients? (multiple choices possible)**		
	Durability in time	67 (78)
	Safety for the patient	67 (78)
	Good clinical results	58 (67)
	Affordable price	54 (63)
	Reliable evidence	55 (64)
	Ease of surgical implementation	43 (50)

**Table 8:** Perceived difficulties with bioprinted cartilage according to place of work (What problems/difficulties do you perceive for its use/work placement? Multiple choices possible)

Difficulty	Surgeons, n (%)	Surgeons in hospitals of low complexity^a^, n (%)	Surgeons in hospitals of medium complexity^b^, n (%)	Surgeons in hospitals of high complexity^c^, n (%)
Uncertainty in results	61 (71)	11 (18)	23 (38)	27 (44)
Authorization by the hospital	50 (58)	11 (22)	16 (32)	23 (46)
Patient safety	46 (53)	10 (22)	16 (35)	30 (65)
Hard to handle	38 (44)	6 (16)	15 (39)	17 (45)
Waiting time	37 (43)	8 (22)	13 (35)	16 (43)
Surgical difficulties	31 (36)	7 (23)	9 (29)	15 (48)

^a^16 surgeons were from hospitals of low complexity (19% of the 86 participants).

^b^34 surgeons were from hospitals of medium complexity (40% of the 86 participants).

^c^36 surgeons were from hospitals of high complexity (42% of the 86 participants).

### Relevant Variables To Use

Once the qualities of the graft were defined, their consequences on the patient’s life were highlighted, from pain reduction to improvement in the quality of life (everyday life satisfaction). Participants were also asked about the need for clinical trials. These data were crossed with the source of information, via any medium or specialized companies, and with the age of the surgeon, as it was previously observed that it was the only relevant demographic variable (Table 9) [23].

No significant differences were observed in terms of the importance of pain reduction, which was considered by all participants as a necessary requisite. Surgeons who had received information via any medium were more pessimistic regarding the positive effects or positive impact the bioprinted cartilage graft could have on the patients’ quality of life (*P*=.03). Surgeons who had received information via specialized companies were more optimistic than the rest (*P*=.03).

**Table 9:** Correlation of variables for the use of a bioprinting cartilage, the need for clinical trials, and age of surgeons.

Variable			Number of surgeons (%)	Mean (SD)	*Z*^a^	*P* value
**To what extent do you consider the alleviation of the patient's pain one of the main characteristics of the new implant?**						
	**Have you been informed by a specialized company about 3D technology?**				**–0.435**	**.66**
		Yes	20 (23)	8.9 (9.96)		
		No	66 (77)	8.44 (1.83)		
**To what extent do you that this technology could have beneficial effects and/or a positive impact on the quality of life of patients?**						
	**Have you received information, through any means, on the latest progress in 3D printing?**				**2.244**	**.03^b^**
		Yes	60 (70)	7.53 (1.67)		
		No	26 (30)	8.27 (1.25)		
	**Have you been informed by a specialized company about 3D technology?**				**2.237**	**.03^b^**
		Yes	20 (23)	8.45 (1.07)		
		No	66 (77)	7.60 (1.63)		
**To what extent do you think evidence of clinical trials is needed to be able to implement the bioprinting cartilage in daily clinical use?**						
	**Have you received information, through any means, about the latest progress in 3D printing?**				**-0.5**	**.62**
		Yes	60 (70)	8.78 (1.71)		
		No	26 (30)	8.77 (1.53)		
	**Age**				**9.825**	**.007^c^**
		<40 years	28 (33)	8.65 (1.87)		
		40-50 years	20 (23)	8.35 (1.18)		
		>50 years	38 (44)	9.16 (1.58)		

^a^Mann-Whitney *U* test.

^b^*P*<.05.

^c^*P*<.01.

Most of the participants highlighted the need for clinical trials, irrespective of the source of information. When the need for clinical trials and the age of the surgeons were crossed, it was clear (*P*=.007) that the age group >50 years in surgeons considered scientific evidence through clinical trials to be most necessary.

## Discussion

### Recent Research

There are a few studies published on the perspective of orthopedic surgeons on the bioprinting cartilage, since it is not yet on the market, but there is research on 3D printed medical implants [27]. This study presents an overview of the characteristics that implants should have as well as surgeons’ knowledge of the decision-making process and their expectations and requirements, a thorough understanding of which is necessary to facilitate implementation of the new technology. This technological adoption requires a proactive role, both from the point of view of orthopedic surgeons and health policies, since it will represent a change in the decision-making process of surgeons and the coverage of health benefits [28].

Recent studies represent a significant advance in the clinical translation of human cartilage and the appropriate surgical procedure. The focus of the research is on the biofabrication of biomaterials that maintain the biocompatibility and biodegradability of the original cartilage while increasing the efficiency of cell growth [29]. Mohanraj et al [30] suggested that the presence of an inflammatory environment is more likely to jeopardize the in vivo success of repairers of cartilage derived from mesenchymal stem cells. Using these cells, Yamasaki et al [31] examined the regeneration of articular cartilage and subchondral bone in artificial corpses.

Although researchers are moving forward in all fields of cartilage bioprinting, we have not been able to find working groups publishing the issues of implementation, and therefore, knowledge of orthopedic surgeons on this topic is scarce.

In our previous research [24], which identified the barriers and facilitators for the bioprinted cartilage use and this new approach, we validated the conclusion that orthopedic surgeons should receive information of higher quality from reliable sources, thus enabling the implementation of the bioprinted cartilage, and that researchers should consider what surgeons believe the cartilage graft should be like.

### Implications and Explanation of the Findings 

The results of this study show that the information received impacts the decision-making process of orthopedic surgeons in a complex and diverse way, as it depends on several variables: the type and origin of the information and its relevance to their demographic data. Previous research [24] found that the amount and quality of the information received was one of the main barriers for the implementation of new technologies. The sample analyzed here shows that orthopedic surgeons lack the specific knowledge of 3D printing as applied to cartilage (Table 4), where 50% of the survey participants who admit to being informed in an unspecific way have almost zero knowledge about it. In contrast, the 20 participants who had been informed by specialized companies considered themselves both better informed and more accepting of new technologies.

Therefore, it could be argued that specialized companies should work closely with orthopedic surgeons to help them acquire more specialized knowledge [32], as shown in Table 3. Another interesting fact is that specifically informed surgeons are more optimistic about the benefits and positive impact of the bioprinted cartilage graft on the quality of life of patients. The origin of the information impacts the level of acceptance and expectations of new technologies, both of which are required for ensuring a wider and easier implementation [33] and are key factors in finding a possible solution to osteoarthritis and improving the life of patients (Table 9). The only variable that is significantly linked to the level of up-to-date knowledge among surgeons is their age: Surgeons aged over 50 years considered themselves better informed. Quite often, the extrapolation of clinical studies to the real world is obstructed by the lack of knowledge of a key factor—the people who will have to implement it.

The second set of goals was to analyze the factors that would provide the ideal context and qualities of an archetypal bioprinted cartilage graft as well as factors perceived as difficulties. The characteristics listed on the survey were size of the lesion, duration of the graft, and age of the patient (Table 7), although more characteristics could possibly have been added. The ideal lesion size preferred by most surgeons was between 1 and 2 cm. A long durability was the most required quality in a graft, which was at least 5 years by 59% of the participants. It could be assumed that if the intervention were proven to be simple and noninvasive, this requisite would not be as important. Since this information is not available, orthopedic surgeons expect a long duration for grafts, predicting possible future reinterventions. The age of the patient presents some debate, as 50% believe that age over 70 years in patients is a limitation, whereas 42% do not consider age a factor. This could be explained by the increased life expectancy of over 70 years in the population. Further research could determine if this difference is a consequence of uncertainty or if it could change with time and experience (Table 7). Surprisingly, 43 of the 86 surgeons believed that the ease of implantation of the graft was not a decisive factor for its use.

For the perceived difficulties in the use of the bioprinted cartilage graft (Table 8), six options were provided, two of which—outcome uncertainty and patient safety—were emphasized by surgeons. Logically, surgeons need positive results from clinical trials in patients before using this technique. As the other answer suggested, issues regarding the implantation and manipulation of the graft were less important, and although they were mentioned in some cases (as the technique is not known yet), they were rated well below the other issues. It is important to highlight this difficulty from the surgeons’ viewpoint: They try to offer solutions to the perceived difficulties but are not able to visualize the graft. Orthopedic surgeons are constantly learning and using new surgical techniques, and they are used to the learning curve. Therefore, as long as there is clinical evidence of the effectiveness of a technique, surgical difficulties are not a deterrent, because surgeons believe they will learn the technique in time.

It was expected that other difficulties linked to practical aspects, such as the hospital management’s authorization to use the technique and the wait time for the graft, would be linked to the type of hospital. Therefore, in medium-complexity hospitals, authorization is less problematic than either of the two abovementioned aspects: There is not as much bureaucracy involved in medium-complexity hospitals as in a high-complexity hospital, and new technologies are more easily accepted than they are in smaller hospitals. Finally, the need for clinical trials is one of the main difficulties for implementation of the technique (Table 9), as almost all survey participants required clinical evidence (the average, in every case, was higher than 8 on a scale of 0-10). A significant finding was that surgeons who asked for more evidence were aged over 50 years, probably ranked higher in the hospital structure, and had both greater responsibility and more decision-making power.

### Strengths and Limitations

The present study should be interpreted in the context of its limitations. The initial proposal planned to cover the entirety of the Spanish territory, through the Spanish Society of Orthopedic and Traumatology Surgery and the Spanish Society of the Knee, and English-speaking specialists through the International Cartilage Research Society, but it was not possible to receive authorization from these societies to send the survey. Our coverage of only a small population is a big limitation, as is the low response rate. In addition, there could be a bias, since the participants who answered the survey were probably more interested in the application of new technologies. Finally, the Chi-squared test might provide inexact results when the values input are small.

### Conclusions, Recommendations, and Future Directions

The process of decision-making is based on precise information of quality, provided by companies specializing in the medical application of 3D printing. This variable seems essential to the acceptance of new technologies. The ideal graft, as described by surgeons, could provide important insight to researchers, at least in the initial stages of development, to satisfy the expectations of surgeons. Implementation research should focus on two variables: ensuring communication flows from researchers to surgeons and ensuring that the opinions of orthopedic surgeons regarding the qualities and issues of the grafts reach researchers, which would help them implement the bioprinted cartilage graft with success.

## Abbreviations

SCCOT: Catalan Society of Orthopedic and Traumatology Surgery

## References

[1] Marks R (2014). Osteoarthritis and Articular Cartilage: Biomechanics and Novel Treatment Paradigms. AAR.

[2] Steinwachs MR, Engebretsen L, Brophy RH (2012). Scientific Evidence Base for Cartilage Injury and Repair in the Athlete. Cartilage.

[3] Mardones R, Jofré CM, Minguell JJ (2015). Cell Therapy and Tissue Engineering Approaches for Cartilage Repair and/or Regeneration. Int J Stem Cells.

[4] Ozmeriç A, Alemdaroğlu KB, Aydoğan NH (2014). Treatment for cartilage injuries of the knee with a new treatment algorithm. World J Orthop.

[5] Tetteh ES, Bajaj S, Ghodadra NS, Cole BJ (2012). The Basic science and surgical treatment options for articular cartilage injuries of the knee. J Orthop Sports Phys Ther.

[6] Farr J, Cole B, Dhawan A, Kercher J, Sherman S (2011). Clinical cartilage restoration: evolution and overview. Clin Orthop Relat Res.

[7] Kon E, Filardo G, Di Martino A, Marcacci M (2012). ACI and MACI. J Knee Surg.

[8] Bernhard JC, Vunjak-Novakovic G (2016). Should we use cells, biomaterials, or tissue engineering for cartilage regeneration?. Stem Cell Res Ther.

[9] Knutsen G, Isaksen V, Johansen O, Drogset JO, Grøntvedt T, Engebretsen L, Ludvigsen TC, Roberts S, Solheim E, Strand T (2007). A randomized trial comparing autologous chondrocyte implantation with microfracture. Findings at five years. J Bone Joint Surg Am.

[10] Murphy S, Atala A (2016). Regenerative medicine technology: on-a-chip applications for disease modelling, drug discovery and personalized medicine.

[11] Jiang Y, Lin H, Tuan R (2017). Overview: State of the ArtFuture Prospectives for Cartilage Repair.

[12] Lipskas J, Deep K, Yao W (2019). Robotic-Assisted 3D Bio-printing for Repairing Bone and Cartilage Defects through a Minimally Invasive Approach. Sci Rep.

[13] Guzzo RM, Scanlon V, Sanjay A, Xu R, Drissi H (2014). Establishment of human cell type-specific iPS cells with enhanced chondrogenic potential. Stem Cell Rev.

[14] Jiang T, Kai D, Liu S, Huang X, Heng S, Zhao J, Chan BQY, Loh XJ, Zhu Y, Mao C, Zheng L (2018). Mechanically cartilage-mimicking poly(PCL-PTHF urethane)/collagen nanofibers induce chondrogenesis by blocking NF-kappa B signaling pathway. Biomaterials.

[15] Deng Y, Kuiper J (2017). Functional 3D Tissue Engineering Scaffolds, Materials, Technologies, and Applications.

[16] Jessop ZM, Al-Sabah A, Francis WR, Whitaker IS (2016). Transforming healthcare through regenerative medicine. BMC Med.

[17] Jessop ZM, Javed M, Otto IA, Combellack EJ, Morgan S, Breugem CC, Archer CW, Khan IM, Lineaweaver WC, Kon M, Malda J, Whitaker IS (2016). Combining regenerative medicine strategies to provide durable reconstructive options: auricular cartilage tissue engineering. Stem Cell Res Ther.

[18] O'Dowd A (2013). Peers call for UK to harness. BMJ.

[19] Gu BK, Choi DJ, Park SJ, Kim MS, Kang CM, Kim C (2016). 3-dimensional bioprinting for tissue engineering applications. Biomater Res.

[20] Di Bella C, Duchi S, O'Connell CD, Blanchard R, Augustine C, Yue Z, Thompson F, Richards C, Beirne S, Onofrillo C, Bauquier SH, Ryan SD, Pivonka P, Wallace GG, Choong PF (2018). In situ handheld three-dimensional bioprinting for cartilage regeneration. J Tissue Eng Regen Med.

[21] Onofrillo C, Duchi S, O'Connell CD, Blanchard R, O'Connor AJ, Scott M, Wallace GG, Choong PFM, Di Bella C (2018). Biofabrication of human articular cartilage: a path towards the development of a clinical treatment. Biofabrication.

[22] Lobb R, Colditz GA (2013). Implementation science and its application to population health. Annu Rev Public Health.

[23] Peters DH, Adam T, Alonge O, Agyepong IA, Tran N (2013). Implementation research: what it is and how to do it. BMJ.

[24] Salvador Verges A, Fernández-Luque L, Yildirim M, Salvador-Mata B, Garcia Cuyàs F (2019). Perspectives of Orthopedic Surgeons on the Clinical Use of Bioprinted Cartilage: Qualitative Study. JMIR Biomed Eng.

[25] Spiegel M, Stephens L, Mc Graw Hill (2007). Schaum's outline of theory and problems of statistics.

[26] Eysenbach G (2004). Improving the quality of Web surveys: the Checklist for Reporting Results of Internet E-Surveys (CHERRIES). J Med Internet Res.

[27] Belvedere C, Siegler S, Fortunato A, Caravaggi P, Liverani E, Durante S, Ensini A, Konow T, Leardini A (2019). A new comprehensive procedure for custom-made total ankle replacements: Medical imaging joint modeling prosthesis design, and 3D Printing. J Orthop Res.

[28] Woltz S, Krijnen P, Pieterse A, Schipper I (2018). Surgeons' perspective on shared decision making in trauma surgery. A national survey. Patient Educ Couns.

[29] Saroia J, Yanen W, Wei Q, Zhang K, Lu T, Zhang B (2018). A review on biocompatibility nature of hydrogels with 3D printing techniques, tissue engineering application and its future prospective. Bio-des Manuf.

[30] Mohanraj B, Huang A, Yeger-McKeever M, Schmidt MJ, Dodge GR, Mauck R (2018). Chondrocyte and mesenchymal stem cell derived engineered cartilage exhibits differential sensitivity to pro-inflammatory cytokines. J Orthop Res 2018 Nov.

[31] Yamasaki A, Kunitomi Y, Murata D, Sunaga T, Kuramoto T, Sogawa T, Misumi K (2018). Osteochondral regeneration using constructs of mesenchymal stem cells made by bio three-dimensional printing in mini-pigs. J Orthop Res.

[32] Davies P, Walker AE, Grimshaw JM (2010). A systematic review of the use of theory in the design of guideline dissemination and implementation strategies and interpretation of the results of rigorous evaluations. Implement Sci.

[33] Zhou G, Jiang H, Yin Z, Liu Y, Zhang Q, Zhang C, Pan B, Zhou J, Zhou X, Sun H, Li D, He A, Zhang Z, Zhang W, Liu W, Cao Y (2018). In Vitro Regeneration of Patient-specific Ear-shaped Cartilage and Its First Clinical Application for Auricular Reconstruction. EBioMedicine.

